# Nontypable *Haemophilus influenzae* Septicemia and Urinary Tract Infection Associated with Renal Stone Disease

**DOI:** 10.2174/1874285801812010243

**Published:** 2018-07-31

**Authors:** Marianne Stærk, Sara A. Tolouee, Jens J. Christensen

**Affiliations:** 1 Department of Clinical Microbiology, Slagelse Hospital, Slagelse, Denmark; 2 Department of Urology, Zealand University Hospital Roskilde, Roskilde, Denmark; 3 Institute of Clinical Medicine, University of Copenhagen, Copenhagen, Denmark

**Keywords:** *Hemophilus influenzae* infection, Invasive disease, Urinary tract infection, Renal stones, Non-typable, Antibiotic resistance

## Abstract

**Introduction:**

*Haemophilus influenzae* commonly causes upper respiratory tract infections and has only rarely been reported etiology of urinary tract infections. Since the introduction of the *Haemophilus influenzae* b (Hib) vaccine, non-typable *haemophilus* species now cause the majority of invasive disease in Europe.

**Case Report:**

We report a case of an adult man with non-typable *Haemophilus influenzae* septicemia, urinary tract infection and bilateral renal stone disease. The patient presented with right sided flank pain and a CT scan showed bilateral renal stones and a right sided ureteral stone causing obstruction.

**Results and Discussion:**

*Haemophilus influenzae* was identified in blood and urine and despite a tendency of increasing antibiotic resistance among *Haemophilus influenzae*, our strain was susceptible to all antibiotics tested. Treatment consisted of 3 days of intravenous cefuroxime, insertion of a right sided JJ ureteric stent and 5 days of peroral ciprofloxacin after discharge. Physicians and microbiologists should be aware of *Haemophilus influenzae* as a possible urinary tract pathogen, especially when urinary tract abnormalities are present, and take the risk of antibiotic resistance into consideration at initial treatment.

## 1 INTRODUCTION

*Haemophilus influenzae* is reported as a rare urinary tract pathogen in adults [1-5]. It is a small gram-negative rod and part of the normal bacterial flora in the human upper respiratory tract; strains may possess a polysaccharide capsule or be non-capsulated. The capsular strains are divided into six serotypes (a-f) based on their polysaccharide capsule whereas the non-capsular lack the polysaccharide capsule and are often less virulent. *H. influenzae* species usually causes upper respiratory tract infections, otitis media, pneumonia and exacerbation of chronic obstructive pulmonary disease. Less frequently, they give rise to invasive disease such as sepsis and meningitis [6, 7]. Here, we report a case of septicemia with a non-capsulated *H. influenzae* strain isolated from blood and urine in a 32-year old man with bilateral renal stones.

## 2 CASE REPORT

A 32-year old man was admitted to a Danish hospital due to right sided flank pain of four days duration. He had no confirmed medical diagnoses, but had previously been tested for Sarcoidosis, Polycythemia vera, stroke and acute coronary syndrome. He also had a history of former steroid-use. The available medical records did not state his vaccination status or previous childhood infections. At hospitalization, he presented with intermittent right sided flank pain, turning into constant pain of VAS 7-8 and radiating to the right side groin. Additional symptoms were nausea, chills, and observation of blood in the urine. Physical examination revealed right sided abdominal and renal pain and a temperature of 38.0 degrees Celsius. His urine tested positive for leucocytes, erythrocytes, nitrite and protein 1 g/L and blood samples showed normal urate levels, elevated ionized calcium levels 1.56 mmol/L, creatinine 122 µmol/L, leukocytosis of 15.9 x 10^9^ /L and CRP 6.4 mg/L increasing to 172 mg/L the next day. CT scan showed bilateral nephrolithiasis as well as right side ureterolithiasis causing obstruction Fig. (**1**). Direct microscopy on three out of three blood culture bottles revealed small gram-negative pleomorphic rods within 24 hours of incubation. Mass spectrometry (Bruker Daltonics using MBT Compass software version 4.1 that contains 6903 MSP´s) identified the strain as *H. influenzae* with a score of 2.24. Microbiology testing of urine routinely cultured on a 5% blood agar plate and a UTI chrome agar plate showed 10^5^ growth of *H. influenzae* confirmed by MALDI-TOF MS (score of 2.15). The strain was found to be a non-capsulated biotype II, susceptible to all antibiotics tested by disc diffusion: penicillin (1 unit, zone diameter: blood = 15 mm, urine = 15 mm), amoxicillin-clavulanate (3 µg), ampicillin (10 µg), ciprofloxacin (5 µg, zone diameter: blood = 35 mm, urine = 41 mm), cefuroxime (30 µg) and piperacillin-tazobactam (36 µg) using EUCAST disc diffusion recommendations. After microbiology samples had been collected, the patient started antibiotic treatment with intravenous ampicillin 1 g x 4 daily and a right sided JJ ureteric stent was surgically inserted. The patient received two doses of ampicillin, but due to subjective discomfort, treatment was changed to cefuroxime 1500 mg x 3 daily. After three days, the patient was discharged with 5 days of peroral ciprofloxacin 500 mg x 2 and a scheduled ambulant stone-removal surgery.

## 3 DISCUSSION

*H. influenzae* Invasive Bacterial Disease (IBD) has especially been related to encapsulated strains, mainly serotype b strains causing septicemia, meningitis or epiglottitis. After introduction of Hib vaccination in the childhood vaccine program, episodes with meningitis and epiglottitis have declined to very few cases annually in Denmark [8]. Invasive disease caused by encapsulated strains belonging to other serotypes do occur. However, there is an increasing trend of infections due to non-capsular strains. Hib vaccination does not give protection against infection with such strains. The latest Epidemiological Report, made by the European Centre for Disease Prevention and Control (ECDC), states that in 2014, 2799 confirmed *H. influenzae* IBD cases were reported (29 countries) [8]. When dividing by serotype it was found that by far the greatest number of cases were caused by non-capsular strains in all age groups (1706 cases, 20 countries) [8]. The highest rates of invasive disease were in children < 1 year old and the elderly with the age of > 65 years, thus, age groups more prone to infectious disease [8]. The most common clinical presentation in this post-Hib vaccine era is pneumonia and septicemia [8, 9]. Our case however was a young immunocompetent man with a urinary tract infection and septicemia. Seldomly, cases of *H. influenzae* invasive disease with recognized foci outside the respiratory tract occurs. *H. influenzae* has been reported as the causative agent of acute pyelonephritis [2, 3], chronic prostatitis [10], endometritis [11] and the *H. influenzae* species has been isolated from urethra in men with symptomatic urethritis [4]. During a 15-year study period, Christensen J.J. *et al.* isolated *Haemophilus* species from different unusual sites such as gynaecological or gastrointestinal sites as well as soft tissue and bone and muscle tissue [12]. Specimens from 80 patients were included and 17 isolates were in pure culture. Nine of these were from the Bartholin’s glands, salpinges or parametrium.

In this case, non-typable *H. influenzae* biotype II was isolated from blood and urine and associated with renal stone disease in a young male patient. He had no respiratory symptoms or alternative focus. Usually, urinary tract infections are caused by *Enterobacteriaceae* such as *E.coli*, *Klebsiella*, *Proteus* and *Serratia* species, and less frequently *Pseudomonas aeruginosa*. Fujii *et al.* recently reported a similar case of a 46-year old man with biotype III bacteremic pyelonephritis and unilateral ureteral stone [3]. In children, *H. influenzae* urinary tract infections are rare and possibly associated with abnormalities of the urinary tract [13, 14]. A retrospective study, over a 24-year period, found 36 children out of 5000 urinary tract episodes, with *H. influenzae* or *H. parainfluenzae* bacteriuria, equivalent to < 1% of the episodes, and the majority of the children had a urinary tract abnormality and/or renal damage [13]. Thus, we argue that *H. influenzae* urinary tract infections could be associated with urinary tract or renal disease in adults, as well as children. A proposed link between stone disease and *H. influenzae* bacteriuria could be the urease activity of the bacteria resulting in ammonia synthesis. This increases the pH of the urine and possibly creates a more favorable environment for bacterial growth as well urolithiasis formation [15]. In addition, it has been demonstrated that non-typable *H. influenzae* from clinical isolates are able to form biofilm [16] which may increase the risk of chronic infection, facilitate further urolithiasis and increase the resistance to antibiotic treatment.

The isolated strain in our case was susceptible to all antibiotics tested with no recognized resistance mechanisms present. However, the emerging antibiotic resistance in recent years against ampicillin and several other antibiotics among strains of *H. influenzae* gives cause for serious concern [17, 18]. Epidemiological studies indicated that 8% to 30% of isolates in Europe and North America, and up to around 50% of isolates in East Asia were resistant to ampicillin [17-19]. The recognized resistance mechanisms are beta-lactamase production and PBP3 alterations by mutation [17, 20]. A Korean study of 540 isolates from respiratory tract infections, predominantly non-typable, showed ampicillin resistance of 58% and beta-lactamase production in 52.4% of the isolates [17]. In Canada, a study of 882 isolates of invasive disease demonstrated an increase in beta-lactamase production from 2007 to 2014 and an increasing trend of significant PBP3 mutations [20]. Therefore, when recognizing a serious invasive infection caused by a *H. influenzae* strain the possibility of eventual antibiotic resistance has to be taken into consideration in the initial treatment recommendation.

Growth of *H. influenzae* requires growth factor X (heme) and factor V (NAD). For their detection, it is crucial using culture methods accommodating these needs, which is not always done when examining urine specimens. This may lead to an underestimation of occurrence of *H. influenzae* in urine samples. In our case identification was done using MALDI-TOF mass spectrometry. In a recent study on identification of respiratory pathogens, including 64 *H. influenzae* strains, by conventional phenotypic methods and mass spectrometry [21], it was shown that mass spectrometry provides identification of these bacteria faster and in a more reliable way than those based on conventional phenotypical methods [21].

## CONCLUSION

To our knowledge, this is the first European report on non-capsulated *H. influenzae* as etiology of an invasive urinary tract infection associated with renal stones in an immunocompetent young adult. In this post-Hib vaccine era, non-capsulated *H. influenzae* strains causes the majority of invasive *H. influenzae* infections. *H. influenzae* may be an underestimated cause of urinary tract infections and should be kept in mind in culture-negative urinary tract infections, especially when urinary tract abnormalities are present. The emerging antibiotic resistance in recent years against ampicillin and several other antibiotics among strains of *H. influenzae* has to be taken into consideration when determining initial antibiotic treatment of invasive disease.

## Figures and Tables

**Fig. (1):**
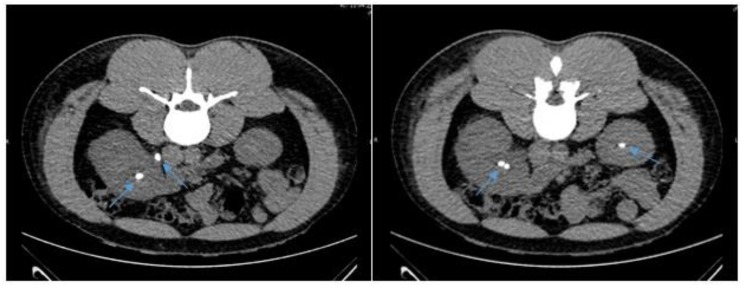
Computed Tomography (CT) scan showing bilateral renal calculi and right sided ureteral calculus (arrows).
